# Skin Performance of Innovative NaDES-Based Gels: In Vivo Evaluation of Anti-Irritation Potential and Short-Term Efficacy

**DOI:** 10.3390/gels11110869

**Published:** 2025-10-30

**Authors:** Milica Martinović, Ivana Nešić, Vanja M. Tadić, Ana Žugić, Marija Tasić-Kostov, Slavica Blagojević, Tomislav Tosti

**Affiliations:** 1 Department of Pharmacy, Faculty of Medicine, University of Niš, Boulevard Dr. Zorana Djindjića 81, 18108 Niš, Serbia; ivana.nesic@medfak.ni.ac.rs (I.N.); marija.tasic.kostov@medfak.ni.ac.rs (M.T.-K.); 2 Department for Pharmaceutical Research and Development, Institute for Medicinal Plant Research “Dr. Josif Pančić”, Tadeuša Koscuška 1, 11000 Belgrade, Serbia; vtadic@mocbilja.rs (V.M.T.); azugic@mocbilja.rs (A.Ž.); 3 Department of Physical Chemistry and Instrumental Methods, University of Belgrade—Faculty of Pharmacy, Vojvode Stepe 450, 11221 Belgrade, Serbia; slavica.blagojevic@pharmacy.bg.ac.rs; 4 Institute of Chemistry, Technology and Metallurgy—National Institute of the Republic of Serbia, University of Belgrade, Studentski Trg 12–16, 11158 Belgrade, Serbia

**Keywords:** NaDES, bilberry leaves, bilberry fruits, green tea leaves, anti-irritation, TEWL, hydration, skin pH, skin biophysical parameters, FTIR

## Abstract

Natural deep eutectic solvents (NaDES) represent novel biodegradable green extraction solvents obtained from natural metabolites such as sugars and organic acids. NaDES-based extracts have demonstrated better performance in in vitro assays compared to those obtained using conventional solvents. In this study, extracts of bilberry leaves (BL), bilberry fruits (BF), and green tea leaves (TL) were prepared using the following NaDES, respectively—malic acid + glycerol (MG), citric acid + sorbitol (CS), and tartaric acid + sorbitol (TS), whose formation was confirmed via FTIR spectroscopy. With the aim to evaluate the effect of gels loaded with NaDES extracts on skin biophysical parameters 2 h prior their application, as well as their anti-irritation potential against sodium lauryl sulfate–induced irritation, an in vivo study involving human volunteers was conducted. The results indicated that all extract-loaded gels exhibited notable anti-irritation potential, reducing artificially induced irritation and improving elevated skin parameters including transepidermal water loss (TEWL), erythema index (EI), and pH. The ΔTEWL at CS–BF site was 8.20 ± 0.34, while at TS–TL was 5.63 ± 0.30. The short-term efficacy study revealed increased skin hydration across all treated sites, preservation of skin pH within physiological limits, and reduction in EI at the site treated with TS–TL gel. Further in vivo studies are planned for confirming long-term skin effects.

## 1. Introduction

Plant-derived actives have long been a focus of research in the formulation of dermocosmetic products. Representing a rich source of polyphenolic bioactive compounds such as flavonoids, tannins, anthocyanins, carotenoids, and vitamins, plant extracts may exhibit various dermocosmetic effects, including anti-inflammatory, antioxidant, antibacterial, UV-protective, depigmenting, as well as nourishing, emollient, and moisturizing. Therefore, they are regarded as excellent candidates for incorporation into emollient products and formulations aimed at caring for all skin types, i.e., conditioning the skin, hydrating dry skin, treating acne-prone skin, protecting against environmental stressors, and slowing the aging process [1,2,3].

The main advantages of using plant extracts as cosmetic actives include the following:

- Multifunctionality;
- Improved safety profile compared to synthetic ingredients;
- Compatibility with all skin types;
- Diversity of plant sources;
- Cost-effectiveness (generally less expensive than synthetic ingredients);
- Sustainability, biodegradability, and environmental friendliness [4].

Green tea (*Camellia sinensis* L.) is a widely used botanical raw material rich in polyphenols. Its leaf (*Camelliae sinensis non fermentatum folium*) (TL) has been used for over a thousand years in traditional medicine [5]. Numerous studies have investigated green tea leaf isolates in formulations for their potential anti-aging, UV-protective, antioxidant, and anti-inflammatory effects [6].

*Vaccinium myrtillus* L. (Ericaceae), commonly known as bilberry, is a low-growing shrub indigenous to North America and northern Europe. The fruit (*Myrtilli fructus*) (BF) is rich in phenolic compounds, particularly anthocyanins, which are associated with various health benefits [7]. In terms of skin effects, bilberry fruit extract is traditionally used for its antiseptic and astringent properties and acts as a skin tonic. Studies have shown that it may inhibit tyrosinase and hyaluronidase, making it a promising ingredient in anti-aging dermocosmetic formulations. Numerous studies have confirmed the antioxidant potential of bilberry fruit extract [8].

During industrial processing of bilberry fruit, by-products such as leaves, seeds, and peels are often discarded as waste despite being rich in bioactive compounds. Bilberry leaves (*Myrtilli folium*) (BL) are rich in flavonoids, phenolic acids (especially chlorogenic, caffeic, and protocatechuic acids), tannins, and stilbenes, and contain these compounds in higher concentrations than the fruit [7]. Unlike the fruit, the leaf does not have a monograph in the European or British Pharmacopoeia.

The use of conventional solvents in extraction is perceived as undesirable due to their toxicity, volatility, and flammability. As a result, alternative solvents such as ionic liquids (ILs), deep eutectic solvents (DES), and natural deep eutectic solvents (NaDES) are being developed and studied. NaDES are considered inert, non-toxic, biodegradable, and environmentally friendly. NaDES are prepared similarly to DES, by the combination of components, resulting in a mixture with a melting point lower than each of their individual components. First mentioned in 2011, it was proposed that cellular metabolites like sugars, amino acids, choline, and organic acids form a third liquid phase in living organisms—NaDES, alongside aqueous and lipid phases, capable of dissolving compounds that are poorly soluble in either. This phase may protect living systems under stress, for example, by concentrating antioxidants to protect cell membranes [9].

Key physicochemical properties of NaDES include biodegradability and ease of preparation. Due to their ability to donate/accept protons and form hydrogen bonds, NaDES enhance the solubility of bioactive compounds with diverse characteristics. Properties such as conductivity, density, and viscosity can be tailored by selecting appropriate starting materials. NaDES typically have higher density than water due to van der Waals forces and large ion sizes, and they generally exhibit low conductivity [10]. Other advantages include non-flammability and thermal stability. NaDES have also been shown to improve the stability of plant-derived compounds and enhance their bioactivity, solubility, and bioavailability of extracted phytochemicals [11].

NaDES-based extracts are commonly incorporated into gels, emulsions, emulgels, or other topical formulations, where they not only enhance drug loading and skin penetration but also serve as functional carriers [12]. In addition to their growing use in the pharmaceutical field, NaDES have increasingly been applied in cosmetics, driven by the rising “return to nature” and “clean beauty” trends. Besides all advantages mentioned so far, they comply with green cosmetics certifications such as NATRUE and COSMOS, and can act as an enhancer of bioactive compounds extraction from agrifood waste, a promising renewable source for cosmetic applications [13,14].

In our previous research, we prepared extracts of BF, BL, and TL using natural deep eutectic solvents (NaDES) composed of alpha-hydroxy acids (AHAs) and polyols: malic acid and glycerol (MG), tartaric acid and sorbitol (TS), and citric acid and sorbitol (CS). Our findings revealed that, compared to extracts made with conventional solvents (water and ethanol), NaDES extracts exhibited higher total phenolic and total flavonoid content. HPLC analysis indicated that they are rich in secondary phenolic metabolites, including chlorogenic acid, protocatechuic acid, and epigallocatechin. Moreover, NaDES extract possessed stronger antioxidant activity, which correlated with its total phenolic content [15]. In vitro assays were also used to compare other biological activities, such as anti-tyrosinase, anti-hyaluronidase, and anti-collagenase effects, between NaDES and conventional extracts. The results have shown that NaDES significantly contributed to the enzyme inhibition [16]. In addition, NaDES extracts showed better UV protective potential in vitro and exhibited UV boosting activity [16,17].

Based on these promising in vitro results, we sought to evaluate the in vivo effects of selected extracts on the skin of healthy human volunteers. For this purpose, hydroxyethyl cellulose-based placebo gel (HEC) and gels with NaDES extracts (MG–BL, TS–TL, or CS–BF) were prepared. Hydrogels represent three-dimensional hydrophilic polymer networks, formed through chemical or physical crosslinking. Due to their capability of absorbing large amounts of water while maintaining structural integrity, they have been used in diverse fields such as biomaterials, wound dressings, and cosmetics, especially as drug delivery systems for topical applications. Polysaccharide-based hydrogels, particularly those derived from starch, dextran, chitosan, and cellulose, are of increasing interest due to their biodegradability, biocompatibility, and wide availability, making them promising alternatives to synthetic polymers. Cellulose, a natural linear polysaccharide made of 1,4-β-d-glucopyranosyl units, is especially attractive for biomedical applications. However, its crystalline structure, stabilized by extensive hydrogen bonding, makes it insoluble in water and limits its direct application in hydrogel formulations, which is why semi-synthetic cellulose derivatives have been developed. Hydroxyethyl cellulose, a non-ionic and water-soluble derivative, is a non-toxic, biocompatible, and biodegradable gelling agent suitable for dermocosmetic formulations. This is one of the most commonly used cellulose derivatives, which can act as a coating material, stabilizer, thickener, and rheological modifier in the formulations [18,19,20].

The primary focus of this study was to analyze the formation of selected NaDES (MG, TS, CS) using FTIR spectroscopy, in order to ensure that the solvent system applied in the in vivo studies was structurally consistent and that the observed skin effects can be attributed to the properly formed NaDES-extract system. The second aim was to formulate and characterize gels with NaDES extracts. Finally, the main aim of the paper was to conduct an in vivo study that included a seven-day anti-irritant potential study on artificially induced irritation using sodium lauryl sulfate (SLS) as well as a short-term two-hour efficacy study, to evaluate in vivo effects on the skin of extracts whose in vitro potential was well established in previous studies.

## 2. Results and Discussion

### 2.1. Characterization and FTIR Analysis of Prepared NaDES

All prepared NaDES were clear, viscous, colorless, and transparent liquids. The selected NaDES formulations were composed of components naturally occurring in biological systems. While malic, tartaric, and citric acids are AHAs commonly found in plants, glycerol and sorbitol are sugar alcohols, i.e., natural polyols present in living organisms. In addition, glycerol, as a non-toxic raw material, is widely used in the production of cosmetic products. Malic and citric acids are also involved in the Krebs cycle [21,22].

Hydrogen bonding plays a key role in the formation of NaDES, based on a hydrogen bond donor (HBD) and a hydrogen bond acceptor (HBA) interaction, leading to charge delocalization. Experimental confirmation of eutectic mixture formation is often challenging, and FTIR spectroscopy is one of the most commonly employed methods for this purpose [23].

MG, TS, and CS represent NaDES formulations obtained by combining α-hydroxy acids and sugar polyols. The presence of hydroxyl and carboxyl groups in these molecules enables the formation of sufficiently strong hydrogen bonds [23].

**Figure 1:**
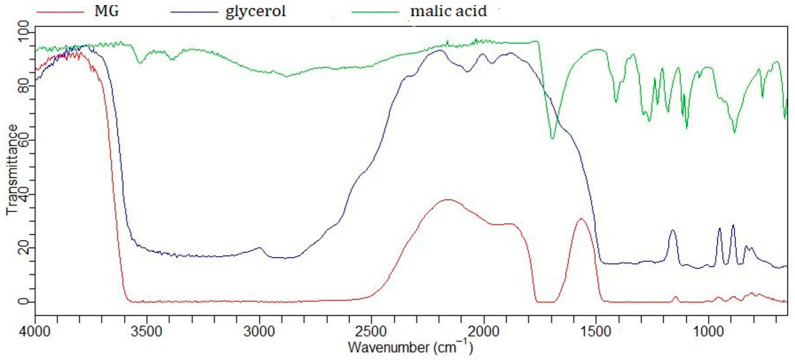
FTIR spectra of malic acid, glycerol, and NaDES formed between them (MG).

Malic acid contains two carboxyl groups and one hydroxyl group, while glycerol is a hydrophilic polyol, allowing for the formation of strong hydrogen bonds between these molecules. In the FTIR spectrum of malic acid (Figure 1), characteristic vibrations of the carbonyl group are observed at 1688 cm^−1^, and of the hydroxyl group at 3523 cm^−1^. Both peaks are altered upon the formation of NaDES. The spectrum of glycerol shows a broad peak corresponding to O–H stretching vibrations in the region of 3100–3500 cm^−1^, which becomes broadened and shifted upon NaDES formation, indicating intermolecular interactions and hydrogen bond formation [23].

Due to its functional groups, glycerol acts as a hydrogen bond donor (HBD), while malic acid can function as both a hydrogen bond donor (HBD) and acceptor (HBA). This NaDES combination has already been investigated in several studies, where its formation was also confirmed using ATR-FTIR spectroscopy [23,24]. In other studies, malic acid has also been successfully combined with other polyols such as sorbitol, xylitol, and propylene glycol for the purpose of NaDES formation [23,25,26].

A similar principle applies to the eutectic mixtures labeled TS and CS. Tartaric acid (two carboxyl groups and two hydroxyl groups) and citric acid (three carboxyl groups and one hydroxyl group) were combined with sorbitol (six hydroxyl groups). Previous studies have shown that citric acid, in addition to sorbitol [27], has also been successfully combined with glycerol [28]. In these NaDES systems, sorbitol, as a polyol, acts as an HBD, while the AHAs may function as both an HBD and HBA.

**Figure 2:**
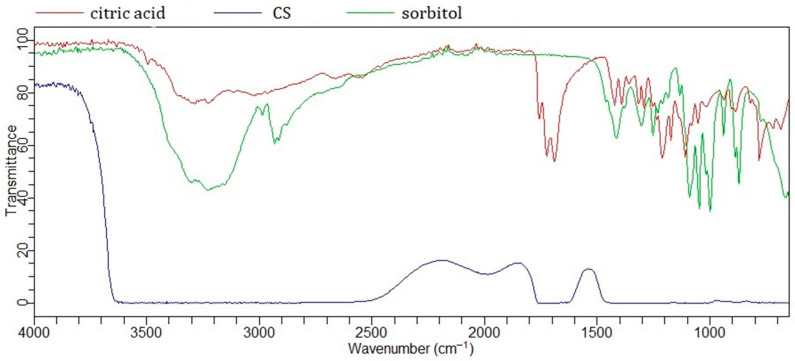
FTIR spectra of citric acid, sorbitol, and NaDES formed between them (CS).

**Figure 3:**
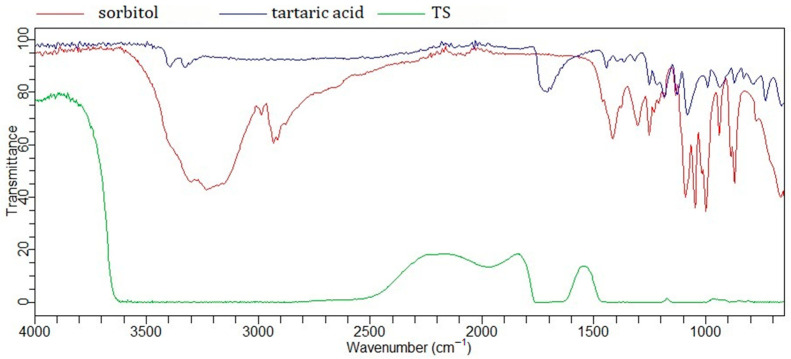
FTIR spectra of tartaric acid, sorbitol, and NaDES formed between them (TS).

In addition to its carboxyl groups, citric acid contains a free hydroxyl group (3298 cm^−1^), whose peak shifts and changes upon NaDES formation (Figure 2) [23]. Changes in the vibrational peaks of the carbonyl groups (1684 cm^−1^, 1725 cm^−1^) indicate interactions with sorbitol. The broad band observed in the FTIR spectrum of CS in the region of 3200–3600 cm^−1^ suggests the formation of hydrogen bonds. Intermolecular interactions are further supported by changes in the broad band of sorbitol (≈3300 cm^−1^), originating from free OH groups, as well as bands in the 2800–3000 cm^−1^ region corresponding to C–H stretching vibrations [29]. The FTIR spectrum of tartaric acid (Figure 3) reveals similar interactions with sorbitol.

The FTIR spectra of NaDES formed between AHAs and polyols appear quite similar, likely due to analogous intermolecular interactions—specifically, hydrogen bonding between C=O and O–H groups. A particularly characteristic feature is the broad O–H band in the 3000–3500 cm^−1^ region, which is also observed in the spectrum of other NaDES spectra, such as the lactic acid–glycine mixture [30].

### 2.2. Physicochemical Characterization of Gels

After confirmation of the NaDES structure, NaDES-based extracts were prepared. Selected NaDES extracts (MG–BL, TS–TL, and CS–BF) were chosen from the results of our previous studies, as the ones with the highest polyphenolic content and the best in vitro measured activities (antioxidant, UV protective, anti-tirosinase, anti-hyaluronidase, anti-collagenase). MG–BL is rich in chlorogenic acid (22.48 ± 1.57 mg/g) and procyanidin B2 (18.59 ± 1.23 mg/g), while TS–TL is abundant in epigallocatechin (32.08 ± 0.84 mg/g) and epigallocatechin gallate (19.18 ± 0.33 mg/g). CS–BF contains mainly chlorogenic acid (1.51 ± 0.01 mg/g) and protocatechuic acid (1.30 ± 0.28 mg/g) [15,16].

**Figure 4:**
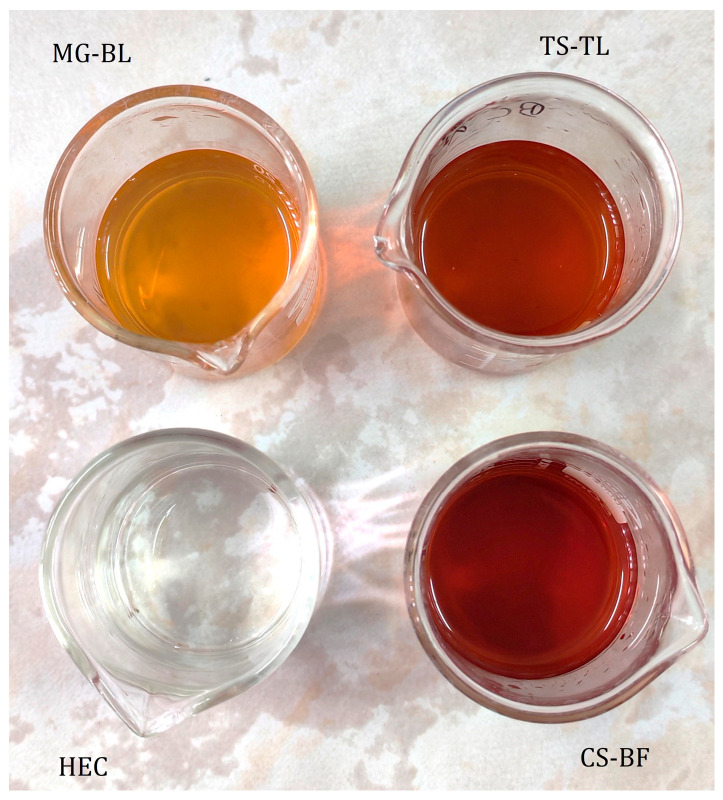
Prepared gels—placebo (HEC) and active gels (MG–BL, CS–BF, and TS–TL).

Selected NaDES-based extracts were incorporated into hydroxyethyl cellulose gels. All prepared gels—placebo (HEC) and active gels (MG–BL, CS–BF, and TS–TL) were transparent, and their color was influenced by the color of the extracts (Figure 4). The pH values of all active gels were adjusted to 4.5 with the addition of sodium hydroxide, in order not to disrupt optimal skin pH during application [31].

Hydroxyethyl cellulose-based gels are known to exhibit non-Newtonian pseudoplastic behavior, characterized by a reduction in viscosity during application, which enables good spreadability [32]. Rheological analysis of placebo HEC and active gels with 12% and 24% of NaDES extracts (combination of MG–BL and TS–TL) conducted in our previous paper confirmed the shear-thinning behavior of gels [17].

The texture properties of a formulation play a crucial role in the optimization of topical preparations, as they directly influence the product’s mechanical characteristics and applicability. These properties include parameters such as hardness, which reflects the ease of application to the skin, and adhesiveness, which indicates the potential retention time at the application site [33]. In the present study, texture analysis was performed to assess adhesiveness, hardness, and cohesiveness of formulated gels (Table 1). The results demonstrated that all gels exhibited similar cohesiveness values. However, the hardness of gels was increased when any of the NaDES extract was added (*p* < 0.05), indicating a decrease in the spreadability of the active gels [34]. This observation was consistent with the measured data for adhesiveness, as all active gels exhibited higher adhesiveness values compared to the placebo gel, suggesting that the NaDES extracts contributed to the prolonged contact of gels to the skin and increased stickiness of the gels. The presence of polyols (glycerol and sorbitol), as components of the NaDES, likely played a role in this enhanced stickiness [35].

The parallel plate method was used as the most common method for measuring the spreadability [36]. Even though the MG–BL gel exhibited the smallest spreading area, which could indicate the lowest spreadability (*p* > 0.05), the results (Table 1) showed no statistically significant differences in the calculated spreading areas of the gels.

### 2.3. In Vivo Evaluation of Biophysical Skin Parameters

An in vivo study was performed to examine the effects of NaDES-based hydrogels on the biophysical parameters of the skin. This investigation was performed using non-invasive methods for measuring skin biophysical properties, including pH, TEWL (transepidermal water loss), EC (electrical capacitance), EI (erythema index), and MI (melanin index), by applying appropriate probes directly to the skin surface.

#### 2.3.1. Anti-Irritant Potential of NaDES-Based Hydrogels

The anti-irritant potential of the plant extracts was confirmed in a seven-day study, in which skin irritation was first artificially induced by topical application of a SLS solution, followed by treatment with active gels (MG–BL, TS–TL, and CS–BF) or a placebo gel without extracts.

Due to its strong cleansing properties and low cost, SLS is commonly found in cosmetic products for hand, hair, and body washing. However, this anionic surfactant can disrupt skin lipid composition and impair the skin barrier function by altering the expression of mRNA markers of keratinocyte differentiation and enzymes involved in corneodesmosome degradation [37]. Therefore, it is frequently used in in vivo studies to induce skin irritation [38,39].

One of the consequences of maintaining physiological skin pH is the preservation of stratum corneum integrity, epidermal barrier homeostasis, and microbiome balance. Changes in pH may occur in certain dermatological conditions or upon contact with chemical agents. Skin pH is also influenced by anatomical location, gender, age, circadian rhythm, and ambient temperature [40]. Some studies have shown that pH increases after the use of soaps and detergents, but returns to physiological levels within 120 min [41]. Irritated skin sites typically show elevated pH values.

**Figure 5:**
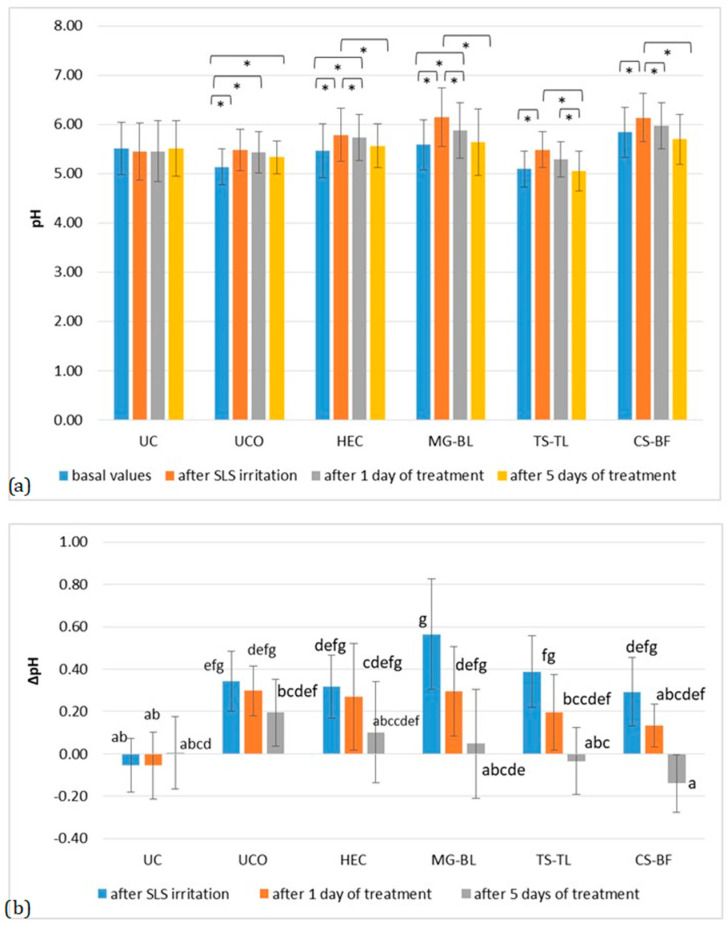
(**a**) Absolute pH values in the in vivo study evaluating the anti-irritant effect of active gels containing selected plant extracts (MG–BL, TS–TL, and CS–BF), as well as a placebo gel without extracts (HEC), an untreated control (UC), and an untreated control under occlusion (UCO). Values are presented as mean ± standard deviation, while statistically significant differences are indicated by the symbol * (*p* < 0.05). (**b**) Absolute ΔpH values (changes relative to baseline values). Statistically significant differences (*p* < 0.05) are marked with different letters.

The results showed that pH increased significantly after irritation (*p* < 0.05), followed by a gradual decrease toward baseline values (Figure 5). At the UCO site, pH values did not return to baseline even five days after irritation, unlike the treated sites. A trend was observed indicating that pH normalized more rapidly at sites treated with active gels. At the site treated with MG–BL gel, despite the highest pH increase due to irritation, a statistically significant reduction in elevated pH was observed after just one day of treatment (ΔpH = 0.27 ± 0.03). At the site treated with TS–TL gel, the reduction was ΔpH = 0.19 ± 0.10, and for CS–BF gel, ΔpH = 0.16 ± 0.10. In contrast, the site treated with placebo gel showed only a minor reduction (ΔpH = 0.05 ± 0.01), indicating the extracts’ role in normalizing disturbed pH values as early as the first day of application. Unlike the placebo site, where the ΔpH after SLS irritation and after five days of treatment did not differ significantly, all sites treated with active gels showed significantly lower pH values at the end of the study compared to post-SLS irritation (*p* < 0.05).

TEWL and EC are commonly used as indicators of skin barrier integrity. An intact skin barrier is essential for preventing dermal absorption of chemicals and infectious agents, which can penetrate only when the barrier is compromised. Moreover, a healthy barrier prevents water loss through the stratum corneum and supports optimal epidermal hydration. Impaired barrier function is characterized by elevated TEWL, a parameter that reflects the rate of water diffusion through the stratum corneum, and is observed in various skin disorders (e.g., atopic dermatitis and psoriasis), as well as in experimental studies where barrier disruption is intentionally induced by solvents and detergents. These agents interact with skin lipids, reducing corneocyte cohesion and skin hydration [42].

**Figure 6:**
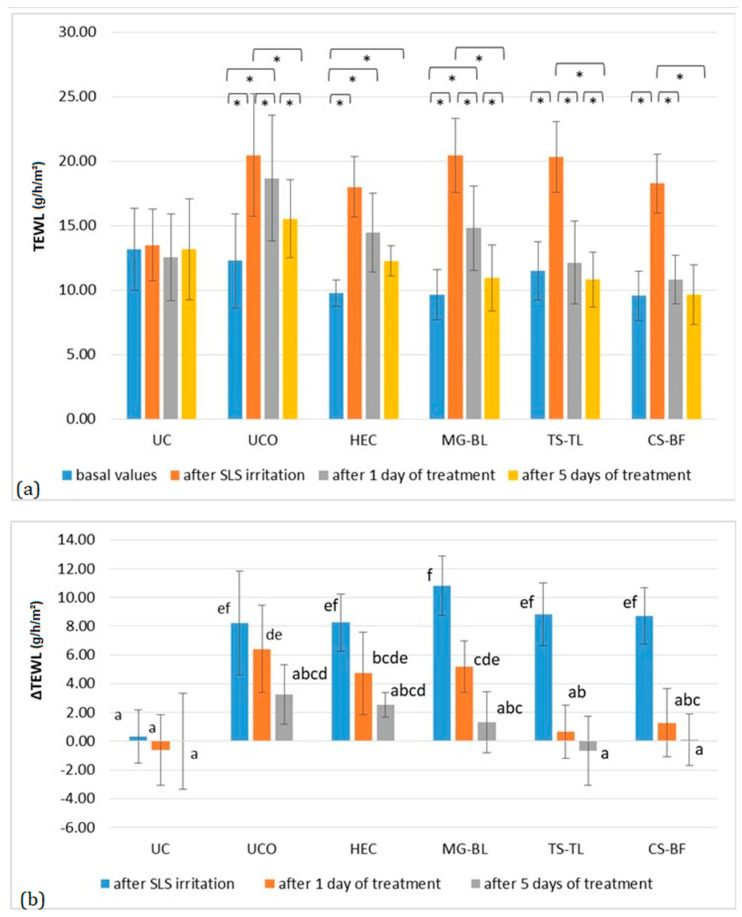
(**a**) Absolute TEWL values in the in vivo study evaluating the anti-irritant effect of active gels containing selected plant extracts (MG–BL, TS–TL, and CS–BF), as well as a placebo gel without extracts (HEC), an untreated control (UC), and an untreated control under occlusion (UCO). Values are presented as mean ± standard deviation, while statistically significant differences are indicated by the symbol * (*p* < 0.05). (**b**) Absolute ΔTEWL values (changes relative to baseline values). Statistically significant differences (*p* < 0.05) are marked with different letters.

All irritated sites showed a statistically significant increase in TEWL (Figure 6), which is a typical skin response to exposure to anionic surfactants [39,41]. Similarly to pH, the restoration of physiological TEWL values occurred at different rates. After one day of treatment, TEWL significantly decreased at all sites treated with active gels (*p* < 0.05), whereas it remained unchanged in the placebo and UC groups. Moreover, post hoc analysis revealed that in the cases of TS–TL and CS–BF, TEWL returned to baseline after just one day, unlike MG–BL and placebo. At the TS–TL site, the reduction in elevated TEWL was ΔTEWL = 5.63 ± 0.30, while at the CS–BF site it was ΔTEWL = 8.20 ± 0.34. After five days of treatment, similar effects were observed for all active gels (*p* > 0.05), indicating that while all formulations demonstrated comparable efficacy in restoring skin barrier function, they acted at different rates, with TS–TL and CS–BF achieving faster barrier recovery than MG–BL.

Adequate skin hydration is crucial for maintaining healthy skin. The stratum corneum contains approximately 20% water, which is partly bound to hygroscopic molecules forming the Natural Moisturizing Factor (NMF), and partly to intracellular keratin. NMF constitutes 20–30% of the dry weight of the stratum corneum and includes amino acids and their derivatives, lactates, urea, and electrolytes. One of the key roles of water in the stratum corneum is its involvement in normal desquamation, mediated by hydrolytic enzymes. When water content falls below a critical level, enzyme function is harmed, leading to corneocyte adhesion and their undesirable accumulation on the skin surface, manifesting as dry, rough, and flaky skin. Skin hydration is assessed indirectly by monitoring electrical properties such as conductance and capacitance, which are directly proportional to water content [42].

**Figure 7:**
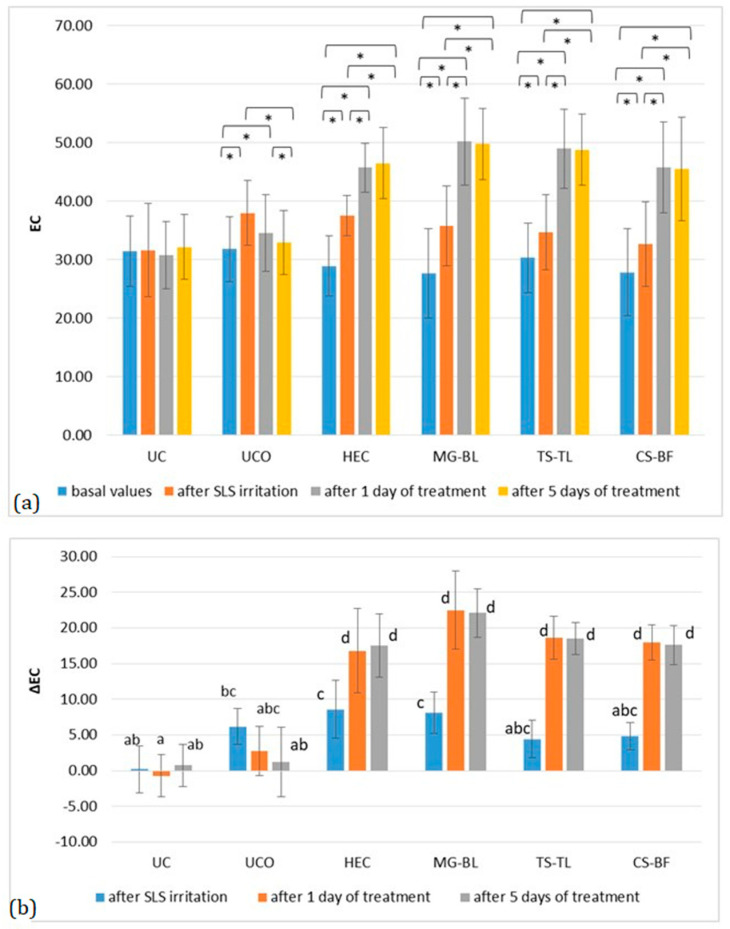
(**a**) Absolute EC values in the in vivo study evaluating the anti-irritant effect of active gels containing selected plant extracts (MG–BL, TS–TL, and CS–BF), as well as a placebo gel without extracts (HEC), an untreated control (UC), and an untreated control under occlusion (UCO). Values are presented as mean ± standard deviation, while statistically significant differences are indicated by the symbol * (*p* < 0.05). (**b**) Absolute ΔEC values (changes relative to baseline values). Statistically significant differences (*p* < 0.05) are marked with different letters.

Changes in EC are shown in Figure 7, where an increase was observed after irritation, followed by a return to baseline at the UC, and a further increase at all treated sites. Hydration increased significantly after just one day of treatment with both placebo and active gels. As shown in Figure 7b, the increase in hydration did not differ significantly between the placebo and active sites. However, a clear trend toward higher hydration at the active sites was observed. Specifically, at the placebo site, ΔEC was 8.20 ± 1.93, while the highest hydration increase was recorded at the MG–BL site (ΔEC = 14.43 ± 2.61), which was nearly twice that observed at the placebo site. Although MG–BL exhibited the highest mean ΔEC value, post hoc analysis showed no statistically significant differences among the active gels, suggesting comparable hydration-enhancing effects across the formulations. The observed increase in skin hydration corresponds with the reduction in TEWL (Figure 6), supporting the hypothesis that NaDES-based extracts enhance skin barrier function by improving both water retention and lipid organization within the stratum corneum.

**Figure 8:**
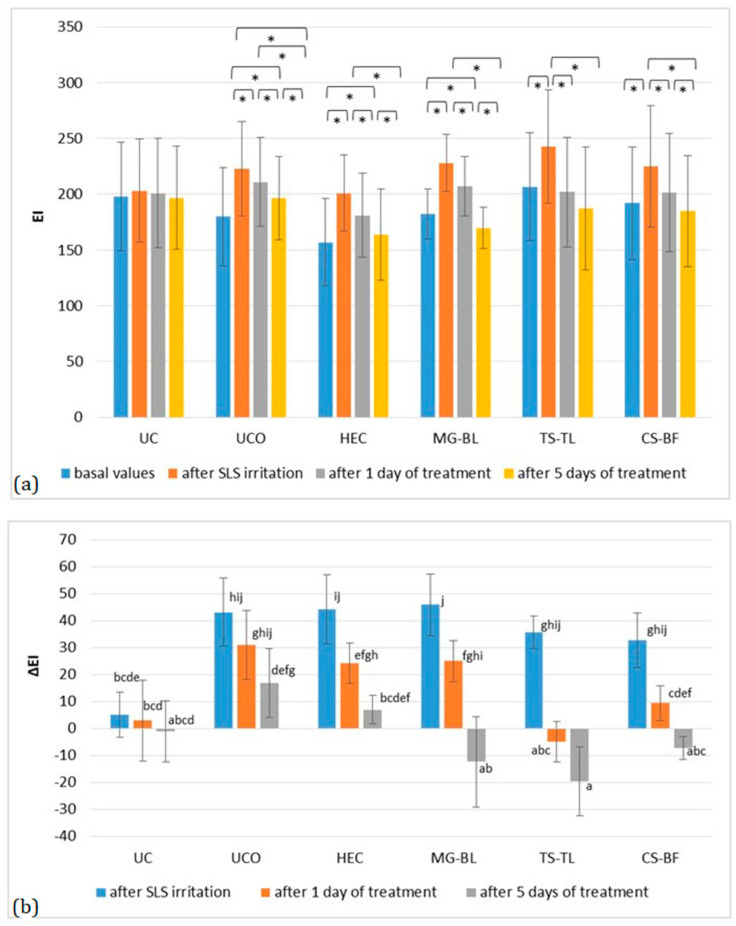
(**a**) Absolute EI values in the in vivo study evaluating the anti-irritant effect of active gels containing selected plant extracts (MG–BL, TS–TL, and CS–BF as well as a placebo gel without extracts (HEC), an untreated control (UC), and an untreated control under occlusion (UCO). Values are presented as mean ± standard deviation, while statistically significant differences are indicated by the symbol * (*p* < 0.05). (**b**) Absolute ΔEI values (changes relative to baseline values). Statistically significant differences (*p* < 0.05) are marked with different letters.

Previous in vitro studies have shown that bilberry fruit and green tea leaf extracts may reduce angiogenesis [43,44], supporting the anti-irritant potential of the plant extracts and the change in EI (Figure 8). All sites showed a significant increase in EI after irritation and removal of occlusion, with an average ΔEI = 29.62 ± 1.79. However, treatment with active gels led to a statistically significant reduction in EI (*p* < 0.05). At the UCO site, a significant decrease occurred only after five days, and even then, values did not return to baseline. At the placebo site, a significant reduction was observed after one day (ΔEI = 12.14 ± 0.2), with values returning to baseline after five days. The most pronounced effect was observed at the TS–TL site, where ΔEI decreased by 40.57 ± 1.55 after one day, while at the MG–BL and CS–BF sites, reductions were ΔEI = 20.86 ± 3.78 and ΔEI = 23.29 ± 3.8, respectively. Notably, differences were observed in the rate of EI reduction among the tested gels. In the case of MG–BL, EI decreased gradually over time (*p* < 0.05 between all measurement points), whereas at the TS–TL and CS–BF sites, no significant differences were found between the first and fifth treatment days, suggesting that erythema at these sites returned to baseline levels more rapidly than with MG–BL. These results indicate that all active gels effectively reduced irritation, although the TS–TL and CS–BF formulations restored normal skin color more rapidly.

Based on all results, it can be concluded that all tested extracts demonstrated anti-irritant activity, reflected in the reduction in elevated TEWL, EI, and pH values caused by irritation. This effect was most pronounced with the TS–TL extract, which restored the measured parameters to baseline values after just one day of treatment.

#### 2.3.2. Short-Term Efficacy of NaDES-Based Hydrogels

In the short-term efficacy of NaDES-based hydrogels, two measurements were conducted. Baseline skin parameter values were measured for each participant, followed by measurements taken two hours after gel application.

**Figure 9:**
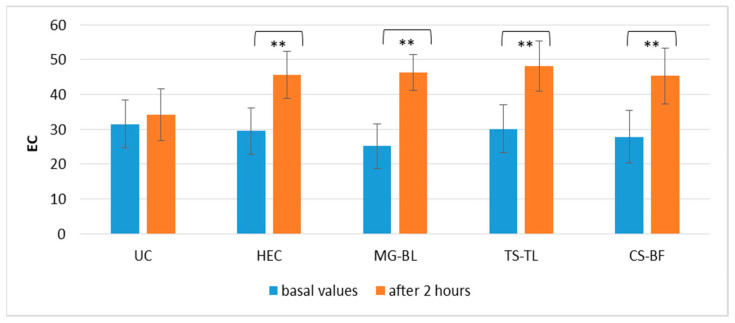
Absolute EC values in the in vivo study evaluating the effects of short-term application of active gels containing selected plant extracts (MG–BL, TS–TL, and CS–BF), as well as a placebo gel without extracts and an untreated control (UC). Values are presented as mean ± standard deviation, while statistically significant differences are indicated by the symbol ** (*p* < 0.01).

The results demonstrated a statistically significant increase in skin hydration at all treated sites (*p* < 0.05) (Figure 9). Although hydration increased more prominently at sites treated with active gels—ΔEC(MG–BL) = 21.17 ± 4.02; ΔEC(TS–TL) = 18.09 ± 3.15; ΔEC(CS–BF) = 17.48 ± 5.04, compared to the site treated with placebo gel (HEC), ΔEC(HEC) = 16.05 ± 3.99, this difference was not statistically significant (*p* > 0.05). This result suggests that short-term application does not result in a significant difference in hydration between active and placebo gel, but indicates the potential for long-term application of active gel to significantly enhance skin hydration. Glycerol, which is commonly used as a humectant in gel formulations to prevent drying and syneresis, can also significantly enhance stratum corneum hydration due to its hygroscopic nature, its interaction with stratum corneum structures, and its “bulking” effect, characterized by intracellular expansion within or between corneocytes [45]. This likely explains the significant increase in hydration observed in all treated sites (placebo and active). As shown in Figure 7, repeated application of the active gels tended to produce a more pronounced increase in hydration compared to the placebo gels. This suggests that prolonged exposure and repeated application may be required for the full manifestation of the extract’s effects [46]. NaDES are known to enhance skin penetration of active components, improve the biological activity of extracts, and serve as effective delivery systems. In addition, due to their hydrogen-bonding capacity, NaDES components can interact with stratum corneum constituents. The composition of NaDES may play a key role in these effects [13]. The selected NaDES extracts are primarily composed of polyols and AHAs, which both can significantly enhance hydration. Polyols exhibit a strong water-binding capacity due to their multiple hydroxyl groups, while AHAs improve skin hydration, smoothness, and tone through several mechanisms [35,47].

Regarding pH values, no statistically significant changes or disruption of physiological skin pH were observed following short-term gel application.

**Figure 10:**
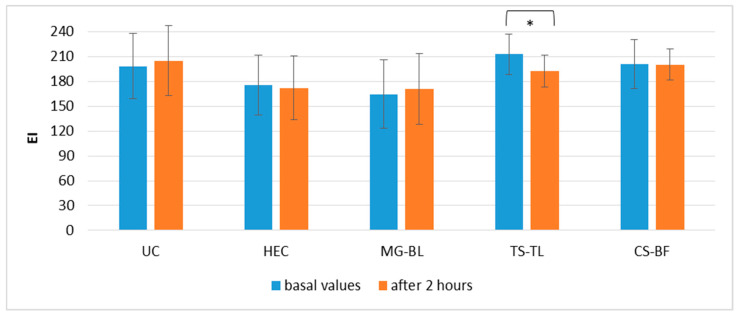
Absolute EI values in the in vivo study evaluating the effects of short-term application of active gels containing selected plant extracts (MG–BL, TS–TL, and CS–BF), as well as a placebo gel without extracts and an untreated control (UC). Values are presented as mean ± standard deviation, while statistically significant differences are indicated by the symbol * (*p* < 0.05).

Similarly, EI values did not change significantly at most sites (Figure 10), except at the site treated with TS–TL gel, where a reduction in EI was observed, ΔEI(TS–TL) = 18.09 ± 3.15, indicating that a decrease in erythema may occur as early as two hours post-application. This finding aligns with previous results from the anti-irritant potential study, which showed that TS–TL gel can reduce EI (Figure 8).

Although the selected extracts contain alpha-hydroxy acids known for their potential to lighten hyperpigmentation, a single application is insufficient to achieve a noticeable skin-lightening effect. The MI parameter, which reflects melanin levels in the skin, did not show statistically significant changes at any of the tested sites, which was expected. Even though significant changes in the melanin index are not expected over a short testing period, its measurement provides supportive information confirming that no unexpected pigmentation changes occurred following the application of the gels.

The results of conducted in vivo studies indicate the potential of compounded gels for dermocosmetic application. The present study was designed as a short-term investigation primarily to provide preliminary data on the efficacy of the NaDES-based formulations. The primary limitation of the research, however, is its brief duration. Therefore, the aim of our future work will be to conduct a 28-day study to confirm the long-term effects of gel application. In addition, the plan for our future work is also to perform a Franc diffusion cell release study as well as a tape-stripping study to quantify the release and skin penetration kinetics of active compounds from the extracts.

## 3. Conclusions

The results of an in vivo study on the determination of the anti-irritation potential of gels with selected NaDES extracts (MG–BL, TS–TL, and CS–BF) showed that the NaDES-based hydrogels successfully normalized all biophysical skin parameters (pH, TEWL, and EI) whose normal values had been disrupted due to artificially induced irritation. The results of an in vivo study on the determination of the short-term effects of the same gels showed that the application of NaDES-based gels led to a significant increase in skin hydration 2 h after their application. In addition, there was a statistically significant reduction in EI at the site treated with the TS–TL gel.

## 4. Materials and Methods

### 4.1. Materials

In this study, the following herbal materials were used: bilberry fruits and bilberry leaves (*Myrtilli fructus*, *Myrtilli folium*, *Vaccinium myrtillus* L., Ericaceae) and green tea leaves (*Camelliae sinensis non fermentatum folium*, *Camellia sinensis* (L.) Kuntze, Theaceae)—the voucher specimens (CSNonF_1121, VMF_1021, and VML_0921), Herbarium of Faculty of Pharmacy, University of Belgrade (Belgrade, Serbia).

For NaDES preparation, AHAs (tartaric acid, malic acid, and citric acid) were purchased from Centrohem (Stara Pazova, Serbia), while polyols (sorbitol and glycerol) were purchased from Comcen (Belgrade, Serbia). For gel preparation, hydroxyethyl cellulose from Chem Point (Krakow, Poland), sodium benzoate and sodium hydroxide from Comcen (Belgrade, Serbia), and purified water from the Faculty of Medicine (University of Niš, Niš, Serbia) were used.

### 4.2. NaDES Preparation

The procedure for NaDES preparation was based on heating and continuously stirring upon melting the two components for 30 min on a magnetic stirrer (IKAMAG, IKA, Verke, Staufen, Germany). The formed NaDES were malic acid—glycerol (1:2 mol/mol) (MG), tartaric acid—sorbitol (1:2 mol/mol) (TS), and citric acid—sorbitol (1:2 mol/mol) (CS) to which distilled water was added at 30% (*v*/*v*) for the purpose of viscosity correction.

### 4.3. FTIR Analysis of Prepared NaDES

To confirm the formation of NaDES, Fourier-transform infrared (FTIR) spectroscopy was used. FTIR spectra of the individual NaDES components, as well as the final NaDES mixtures, were recorded at room temperature using an Agilent Cary 630 FTIR Spectrometer (Agilent Technologies, Inc., Santa Clara, CA, USA) and analyzed with Agilent MicroLab software, version 5.8.

### 4.4. NaDES Extracts Preparation

NaDES extracts were prepared using ultrasound-assisted extraction in a sonication water bath (Gesellschaft fur Labortechnik, Burgwedel, Germany). The dried powdered plant material was mixed with solvents (MG, TS, CS) in a mass ratio of 1:20, a commonly used ratio in the preparation of NaDES extracts [48,49,50]. The extraction was conducted at 50 °C for 30 min, after which the extracts were centrifuged. The supernatant was incorporated into gels.

### 4.5. Gel Preparation

For the purpose of evaluating the anti-irritant potential and short-term efficacy of selected plant extracts, the extracts were incorporated into a hydroxyethyl cellulose gel (Table 2). This approach is commonly employed in in vivo studies investigating the effects of plant extracts on human skin, as gel incorporation facilitates application (especially in cases requiring repeated administration) and prolongs skin contact.

The gels were prepared at room temperature by a standard hydrogel preparation procedure based on dispersing the gelling agent (HEC) in the water phase (water, glycerin, and a preservative) using the RW 16 basic propeller rotary laboratory stirrer (IKA Werke, Staufen, Germany). NaDES extracts were incorporated into the end of each prepared gel.

### 4.6. Physicochemical Gel Characterization

For pH measurements pH meter electrode (pH 211 Microprocessor pH Meter, Hanna Instruments, Woonsocket, RI, USA) was used.

The texture profile analysis (TPA) was performed 48 h after gel preparation, using a CT3 texture analyzer (Brookfield, AMETEK Inc., Middleborough, MA, USA) for measuring the following texture parameters: hardness, cohesiveness, and adhesiveness. Hardness was calculated as the mean value of hardness cycle 1 and hardness cycle 2, measured during both TPA cycles. The trigger load of the cone probe (TA-STF) was set at 20 mN, while its test speed during immersion into the sample was 1 mm/s. The target value was set at 5 mm.

The spreadability was measured using the parallel plate method, based on placing 0.3 mL of gel between two glass plates, 20 × 20 cm, after which a weight of 100 g was placed on the top for 1 min, and the spreading area diameter *d* (mm) was measured. Spreadability, i.e., spreading area (*Si*), was calculated using Equation (1) [36].

(1) $$S_{i}=d^{2}\frac{\pi }{4}$$

### 4.7. In Vivo Study

For the in vivo study conducted on human volunteers, approval was obtained from the Ethics Committee of the Faculty of Medicine, University of Niš (Decision No. 12-10650/2-6, dated 3 October 2022). All procedures were performed following the acquisition of written informed consent from each participant, in accordance with the Declaration of Helsinki and after providing comprehensive information regarding the study protocol.

**Figure 11:**
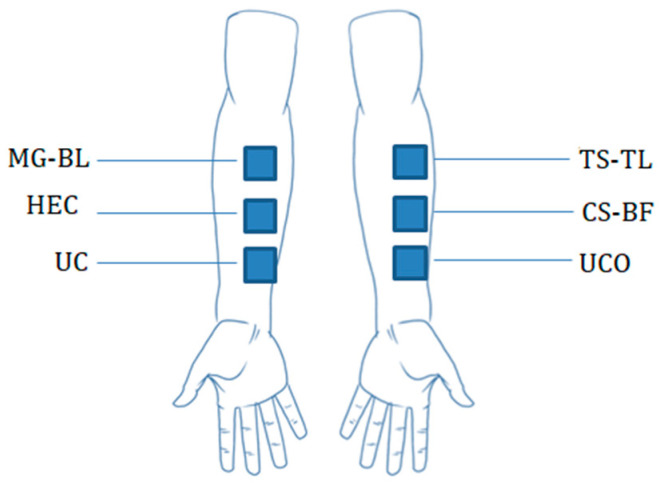
Schematic representation of the volar forearm with marked application site.

The tested gels were labeled with letters indicating the application site (LU—left upper, LM—left middle, RU—right upper, RM—right middle). A cardboard template was used to precisely determine the location and position of gel application (Figure 11).

All study participants were healthy volunteers with no prior history of dermatological conditions. They were instructed to refrain from using any other cosmetic products on the test area of the skin for one week prior to and throughout the duration of the in vivo study.

The in vivo measurements were conducted using the Multi Probe Adapter System MPA^®^ 9 (Courage + Khazaka electronic GmbH, Köln, Germany), comprising the following probes:

- Corneometer^®^ CM 825—for assessing skin hydration (Stratum Corneum Hydration, SCH) by measuring the electrical capacitance of the skin (EC);
- Tewameter^®^ TM 300—for evaluating transepidermal water loss (TEWL) following a device stabilization period of approximately 40 s; the TEWL value with the lowest standard deviation was recorded;
- Mexameter^®^ MX 18—for determining the melanin index (MI) and erythema index (EI), used to quantify skin pigmentation and redness, respectively;
- Skin-pH-Meter^®^ PH 905—for measuring the skin surface pH.

Prior to each study session, all probes were calibrated according to the manufacturer’s instructions. TEWL was expressed in g/h/m^2^, while EC, MI, and EI were expressed in arbitrary units.

The RHT 100 Ambient Condition Sensor was used to monitor room temperature and humidity. Prior to each measurement session, all volunteers spent 30 min with bare forearms in a climate-controlled room (temperature: 22 ± 1 °C; relative humidity: 45 ± 5%) to ensure acclimatization and minimize environmental influence on the measured biophysical parameters.

According to the recommendations of the European Group for Efficacy Measurement of Cosmetics and Other Topical Products (EEMCO) and revised EEMCO guidelines, subjects should ideally acclimatize for at least 20 min at ambient temperature (20–22 °C) and relative humidity (40–60%) [51,52].

#### 4.7.1. Evaluation of the Anti-Irritant Potential of NaDES-Based Gels

The anti-irritant potential of the NaDES-based gels was evaluated in a double-blind study involving 20 healthy volunteers of both genders, with a mean age of 25.85 ± 4.74 years. The study was conducted during the month of November.

Prior to irritation induction (Day 1 of the study), baseline skin parameters were measured, including EC, TEWL, EI (erythema index), and pH. Irritation was then induced by applying a 3 × 3 cm filter paper soaked with 75 µL of a 6% SLS solution to predefined sites on the volar forearm of each participant. These sites were covered with Parafilm^®^ occlusive film (American National Can. Co., Chicago, IL, USA), followed by Omnifix^®^E adhesive tape (Hartmann, Heidenheim, Germany). One site was designated as an untreated control, where no irritation was induced.

After 6 h of occlusion, artificial skin irritation was successfully induced. Participants were instructed to return the following day for measurement of parameters on the irritated skin (Day 2 of the study). At the sites where irritation had been previously induced, gels were applied (either active or placebo gel), while one site was designated as an untreated occluded control (a site where irritation was induced using SLS but no gel was applied afterward).

Participants were instructed to apply the gels twice daily over a period of 5 days. Measurements were conducted on Day 3 (24 h after the beginning of gel application) and Day 7 (after 5 days of application) [53].

#### 4.7.2. Evaluation of the Short-Term Effect of NaDES-Based Gels

The effects of short-term application of NaDES-based gels were evaluated in a double-blind study involving 20 healthy volunteers of both genders, with a mean age of 27.65 ± 6.99 years. The study was conducted during the month of November.

At predefined sites on the volar forearm of each participant, the active gel was gently rubbed into the skin (0.016 g/cm^2^). Two hours after application, skin parameters were measured, including EC, EI, MI, and pH. These values were then compared to the previously recorded baseline measurements (prior to gel application).

### 4.8. Statistical Analysis

The estimation of the required number of volunteers for participation in the study was performed using the G*Power 3.1 software. Based on the input parameters (effect size f = 0.4, which was chosen based on previous internal pilot data; Type I error probability α = 0.05; study power β = 0.8; number of groups = 1; number of measurements = 2 or 4; correlation between repeated measures = 0.5; non-sphericity correction ε = 0.75 for four measurements, ε = 1 for two measurements), it was estimated that a minimum of 15 volunteers were needed for the short-term efficacy assessment of the plant extracts, and a minimum of 12 volunteers for the evaluation of their anti-irritant potential.

The results obtained in the in vivo study were presented as mean ± standard deviation and as absolute changes in measured parameters relative to baseline values at specific time intervals ± standard deviation. Statistical comparisons were performed using one-way ANOVA (results of texture analysis and comparison between tested groups—control, placebo, and active in in vivo study) or repeated-measures ANOVA (within-group comparisons over time in in vivo study) with the appropriate post hoc test (Tukey’s test), with statistical significance set at *p* < 0.05. Comparisons of measured values for the same sample in cases where only two measurements were conducted were performed using Student’s *t*-test. Statistical analysis was carried out using IBM SPSS Statistics 20, while diagrams were created using Microsoft Excel 10.

## Figures and Tables

**Table 1:** Results of texture analysis and spreading area of prepared gels—placebo (HEC) and active gels (MG–BL, CS–BF, and TS–TL). Statistically significant differences (*p* < 0.05) within each row are marked with different letters (a, b, c).

	HEC	MG–BL	CS–BF	TS–TL
Hardness (mN)	930.33 ± 64.08 ^a^	1791.00 ± 52.09 ^b^	1670.67 ± 34.95 ^c^	1699.00 ± 23.26 ^c^
Adhesiveness (mJ)	1.47 ± 0.06 ^a^	1.90 ± 0.10 ^b^	1.93 ± 0.06 ^b^	2.13 ± 0.15 ^b^
Cohesiveness	0.93 ± 0.15 ^a^	0.80 ± 0.10 ^a^	0.94 ± 0.17 ^a^	0.85 ± 0.05 ^a^
Spreading area (mm^2^)	504.49 ± 45.32 ^a^	406.11 ± 79.77 ^a^	680.33 ± 138.46 ^a^	617.53 ± 87.94 ^a^

**Table 2:** Composition of gels used in the in vivo study on the safety and efficacy of plant extracts applied to the skin of healthy volunteers.

INCI Name		Composition (%, *w*/*w*)			
		Active Gel— Gel with NaDES Extract			Placebo Gel Without Plant Extract (HEC)
		MG–BL	TS–TL	CS–BF	
Hydroxyethyl cellulose		2	2	2	2
Glycerol		10	10	10	10
Sodium benzoate		1	1	1	1
NaDES extracts	MG–BL	24	-	-	-
	TS–TL	-	24	-	-
	CS–BF	-	-	24	-
NaOH, 10%		q.s. ad pH 4.5	q.s. ad pH 4.5	q.s. ad pH 4.5	-
Water		q.s. ad 100	q.s. ad 100	q.s. ad 100	q.s. ad 100

## Data Availability

The original contributions presented in this study are included in the article. Further inquiries can be directed to the corresponding author.

## Abbreviations

The following abbreviations are used in this manuscript:

NaDES	Natural Deep Eutectic Solvent
TS	Tartaric acid-sorbitol NaDES
CS	Citric acid-sorbitol NaDES
MG	Malic acid-glycerol NaDES
BF	Bilberry fruit
BL	Bilberry leaves
TL	Green tea leaves
SLS	Sodium lauryl sulfate
EC	Electrical capacitance
TEWL	Transepidermal water loss
EI	Erythema index
MI	Melanin index
FTIR	Fourier-Transform Infrared spectroscopy
AHAs	Alpha-hydroxy acids
